# Impact of ATP synthase/coupling factor 6 in hypoxic pulmonary arterial hypertension: An experimental rat model

**DOI:** 10.55730/1300-0144.5485

**Published:** 2022-07-24

**Authors:** Nannan LI, Yugen SHI, Jie YIN, Li SUN, Qingshan ZHANG, Shuai BAO, Juan ZHANG, Youlei LI, Miaomiao WANG, Yanwei ZHANG, Mei XUE, Lei QI, Yan LI, Suhua YAN, Xiaolu LI

**Affiliations:** 1 Department of Chinese Medicine Ophthalmology, the First Affiliated Hospital of Shandong First Medical University, Shandong, China; 2 Department of Cardiology, The First Affiliated Hospital of Shandong First Medical University & Shandong Provincial Qianfoshan Hospital, Shandong Medicine and Health Key Laboratory of Cardiac Electrophysiology and Arrhythmia, Shandong, China; 3 Department of Emergency Medicine, The First Affiliated Hospital of Shandong First Medical University & Shandong Provincial Qianfoshan Hospital, Shandong Medicine and Health Key Laboratory of Emergency Medicine, Shandong, China; 4 Department of Emergency Medicine, Shandong Provincial Qianfoshan Hospital, Shandong University, Shandong, China; 5 Department of Intensive Care Unit, The First Affiliated Hospital of University of Science and Technology of China, Anhui, China; 6 Department of Emergency, People’s Hospital of Xia Jin, Dezhou, China; 7 Department of Emergency, Shandong First Medical University, Shandong, China; 8 Medical Research Center, Shandong Provincial Qianfoshan Hospital, the First Hospital Affiliated with Shandong First Medical University, Shandong, China

**Keywords:** Coupling factor 6, adenosine triphosphate synthase activity, hypoxic, pulmonary arterial hypertension

## Abstract

**Background/aim:**

Hypoxia-induced pulmonary arterial hypertension (PAH) is characterized by prostacyclin (PGI_2_) disorder, which manifests in the same manner as in monocrotaline (MCT)-induced PAH. Endogenous PGI_2_ inhibitor coupling factor 6 (CF6) is involved in MCT-induced PAH. This study aimed to explore the presence or absence of a correlation between hypoxia-induced PAH and CF6.

**Materials and methods:**

This study was conducted between January 2019 and June 2020. A total of 135 male Wistar rats (aged 8 weeks and weighing 200–250 g) were randomly divided into five groups: (A) control, (B) 1 week of hypoxia, (C) 2 weeks of hypoxia, (D) 3 weeks of hypoxia, and (E) 4 weeks of hypoxia. CF6 expression in both lung tissue and blood samples from the lung vasculature and tail vein was measured by western blotting, immunohistochemistry, reverse transcription polymerase chain reaction, and enzyme-linked immunosorbent assay.

**Results:**

Hemodynamic and morphological changes in hypoxia-induced rats indicated PAH development. The results showed the presence of a correlation between the mRNA and protein levels of CF6 in lung tissue, activity of mitochondrial ATP synthase, and hypoxia time, and there was a significant increment in the group exposed to hypoxia for 4 weeks compared to the control group. The decrement expression of ATPase inhibitory factor 1 (IF 1) mRNA was consistent with the outcomes of ATP synthase activity in lung tissue in the 4 weeks of hypoxia group compared with the control group. However, the levels of CF6 and ATP synthase activity did not differ between blood samples from the lung vasculature and tail vein.

**Conclusion:**

In hypoxia-induced PAH, CF6 showed downregulated expression in lung tissue, but not in pulmonary vasculature and circulation. Therefore, we speculated that CF6 and ATP synthase may play important roles in hypoxia-induced PAH.

## 1. Introduction

Pulmonary arterial hypertension (PAH), a fatal disease with sustained increased pulmonary vascular resistance and abnormal pulmonary vascular remodeling, eventually leads to right-sided heart failure and death. Pathological changes in PAH include destruction of pulmonary vascular cell homeostasis and extensive vascular disease. Over the past several decades, significant progress has been made in the symptomatic relief of PAH, including the use of endothelin receptor antagonists, vasodilators, and phosphodiesterase inhibitors. However, these approaches do not improve the overall survival rate [ 1 ]. Benza et al. demonstrated that the five-year survival rates of patients has remained at 57% [ 2 ]. The high morbidity and mortality caused by PAH are still unacceptable [ 3 ], and new treatment regimens are needed.

The 6th World Symposium on Pulmonary Hypertension proposed that the hemodynamic definition of PAH should be changed to a mean pulmonary artery pressure (mPAP) of > 20 mmHg [ 4 ]. Pathological lesions typically present in PAH patients upon clinical presentation, such as pulmonary artery medial hypertrophy, adventitial thickening, or neointimal proliferation [ 5 ]. However, the mechanisms underlying these pathological lesions are not completely understood. Abnormal proliferation and secretion of endothelial cells in PAH increases the synthesis of vasoconstrictors and decreases the synthesis of vasodilators. PAH patients show enhanced levels of potent vasoconstrictors and impaired synthesis of vasodilatory factors, such as prostacyclin (PGI_2_) [ 6, 7 ].

Coupling factor 6 (CF6), an essential subunit of the stalk of mitochondrial Adenosine triphosphate (ATP) synthase, was recently identified as a novel endogenous inhibitor of PGI_2_ [ 8 ]. This is important because the PGI_2_ pathway is currently a major target for PAH treatment. CF6 can be released into the blood as a circulating vasoconstrictive peptide after vascular endothelial cells experience mechanical forces, stress, hypoxia, or high glucose levels [ 9, 10 ]. In addition, the levels of CF6 have been shown to markedly increase in rats with lung disease [ 11, 12 ]. Our previous experiments have also demonstrated that CF6 was increased in monocrotaline (MCT)-induced PAH and that the inhibition of CF6 alleviated PA remodeling in rats [ 13 ]. These results demonstrated the critical role of CF6 in the pathogenesis of MCT-PAH. Hypoxia-induced PAH is also characterized by PGI_2_ disorder and PA vasoconstriction and remodeling; however, it is mechanically different from MCT-induced endothelial injury. As an endogenous PGI_2_ inhibitor, CF6 may play an important role in hypoxia-induced PAH.

Our previous experiments showed that CF6 was markedly increased in MCT-induced PAH rat models and that the inhibition of CF6 alleviated PA remodeling. The pathophysiological mechanism of PAH caused by MCT differs from that observed in clinical settings. Therefore, we explored the potential role of ATP synthase-CF6 in hypoxia-induced PAH.

## 2. Material and methods

### 2.1. Animal models

The animal study was conducted between January 2019 and June 2020 [ 14 ]. One hundred and thirty-five male Wistar rats (aged 8 weeks and weighing 200–250 g) were obtained from the Beijing Vital River Laboratory Animal Technology Co., Ltd. The rats were housed in a standard animal room at a temperature of 21 ± 1 °C, humidity of 55% ± 5%, and 12-h light/dark cycle with free access to water and food. All experimental protocols and procedures used in this study were approved by the Animal Care Committee of Shandong University Affiliated with Qianfoshan Hospital (protocol number: S 030). This study was in accordance with the “Guidelines for the Care and Use of Laboratory Animals” from the National Institutes of Health (NIH Publications No. 8023, revised 1978).

Rats were randomly assigned to one of five possible groups according to the amount of time under hypoxic conditions: group A (n = 15) was the control group; group B (n = 30) included rats exposed to hypoxia (kept in normobaric hypoxia [10% O_2_] by establishing a set-point of 10% O_2_ in the oxygen controller with N_2_ tank and allowed the system to reach a steady state [XBS-03]) for 1 week; group C (n = 30) included rats exposed to hypoxia for 2 weeks; group D (n = 30) included rats exposed to hypoxia for 3 weeks; and group E (n = 30) included rats exposed to hypoxia for 4 weeks [ 15 ].

All animals were monitored daily until they developed symptoms of PH, such as weight loss and tachypnea. At weeks 1, 2, 3, or 4, animals were anaesthetized using sodium pentobarbital (30 mg/kg) and intubated using a small animal ventilator (HX-300S; Chengdu TME Technology Co., Ltd., China) at a rate of 60 breaths/min and a tidal volume of 1.1–1.3 mL/100 g. Hemodynamic, morphologic, and biochemical assessments were performed.

### 2.2. Echocardiography and hemodynamic measurements

The rats in the experimental groups were anesthetized with an intraperitoneal injection of sodium pentobarbital (30 mg/kg). Room temperature was maintained at approximately 25 °C. Cardiac function was evaluated using a Visual Sonics Vevo 770 echocardiographic machine (Visual Sonics, Toronto, Canada) equipped with a 14-MHz linear transducer. An echocardiographic expert (Y.J.) performed measurements in a blinded manner. Short- and long-axis B-dimensional parasternal views of both ventricles at the level of the papillary muscles were acquired to visualize the left ventricle (LV) and right ventricle (RV). Pulmonary artery pressure transduction was conducted with the right jugular vein using a 1.4-F Millar Mikro-Tip catheter transducer (Millar Instruments Inc., Houston, TX, USA) directed to the main pulmonary artery after insertion into the right ventricular outflow duct, although RV systolic pressure (RVSP) was detected with a power laboratory monitoring device (Miller Instruments). Hemodynamic values were accurately computed the LabChart 7.0 physiological data acquisition system (AD Instruments, Sydney, Australia).

### 2.3. Tissue processing and histology

After performing the above tests, cardiac arrest was induced using an overdose of sodium pentobarbital (160 mg/kg body weight). After thoracotomy, the entire lung was excised. The left lung was removed and frozen in liquid nitrogen, and the right lung was inflated with 0.5% low-melting agarose at a constant pressure of 25 cm H_2_O and then fixed in 10% formalin for 24 h. Next, the heart was isolated. The weight ratio of the right ventricle to the left ventricle plus the septum (RV/[LV + S]) was determined using Fulton’s index [ 16 ].

### 2.4. Western blot

Western blot analysis was performed using a Protein Extraction Kit (Beyotime Institute of Biotechnology, Jiangsu, China) to isolate proteins, and protein concentration was measured using a bicinchoninic acid (BCA) protein assay reagent kit (Pierce) [ 17 ].

The same amount of total protein (60 μg) was applied to each lane of a sodium dodecyl sulfate–polyacrylamide gel electrophoresis (SDS-PAGE) (5%–12%) and then transferred onto a polyvinylidene difluoride (PVDF) membrane. The membranes were then blocked with 5% nonfat dry milk in phosphate buffer solution Tween 20 (PBS, T, containing 0.05% Tween 20), and then incubated overnight at 4 °C with primary antibodies against CF6 (Abcam, MA, USA, 1:1500) and α-smooth muscle actin (α-SMA) (Abcam, MA, USA, 1:1500). Subsequently, blots were developed using an enhanced chemiluminescence (ECL) detection kit (Millipore, Merck, Germany). Images were visualized using a FluorChem E Imager (Protein-Simple, Santa Clara, CA, USA). α-SMA can indicate the severity of pulmonary vascular muscularization. Measurements to determine relative densities were normalized to that of a standard protein (GAPDH) (Proteintech, Wuhan, China) using NIH Image J software.

### 2.5. ELISA

After high-dose pentobarbital administration, the blood samples obtained from the tail vein and pulmonary vasculature were stored at room temperature for 1 h and were then centrifuged at 3000 rpm at 4 °C for 15 min. Plasma samples were collected and stored at −80 °C. Lung tissue from the left lung was kept at 4 °C for 12 h and then at room temperature for 1 h. Lung tissue was rinsed with normal saline. After grinding, the homogenate was adjusted using PBS at a rate of 10%. The supernatant was centrifuged at 5000 × g at 4 °C for 15 min. CF6 levels in blood samples from the tail vein and pulmonary vasculature were measured using enzyme-linked immunosorbent assay (ELISA) performed with a commercial enzyme immunoassay kit (EIA Assay Design, Inc.; Ray Biotech, Norcross, GA) according to the manufacturer’s instructions. The amount of CF6 was expressed in picograms per milliliter of protein.

### 2.6. Determination of mitochondrial ATP synthase activity

Mitochondria were isolated from rat lung tissues using a mitochondrial isolation kit (Abcam, MA, USA, ab110168). ATP synthase activity was assayed spectrophotometrically using a microplate assay kit (Abcam, MA, USA, #ab109714) according to the manufacturer’s instructions.

### 2.7. Reverse transcription polymerase chain reaction (RT-PCR)

ATPase inhibitory factor 1 (IF1) is a physiological endogenous inhibitor of ATP synthase. IF1 inhibits the synthetase and hydrolase activities of ATP synthase. Many recent studies have shown that IF1 plays an important role in stabilizing the structure of ATP synthase in normoxic environment [ 18–20] . To examine whether the changes in IF1 and ATP synthase in hypoxic environments are the same, we measured the mRNA expression level of IF1 in lung tissue. Total RNA was extracted from the lung tissues using TRIzol reagent (Invitrogen, Germany). cDNA was synthesized from 1 μg RNA using a Prime Script RT Reagent Kit (Vazyme, Nanjing, China), as described previously. mRNA expression was determined using gene-specific primers and SYBR Green (SYBR Green Gel Dye) using a Bio-Rad iQ5 Multicolor Real-Time PCR machine (Bio-Rad Laboratories, USA). For each sample, glyceraldehyde-3-phosphate dehydrogenase (GAPDH) and the target gene were amplified in triplicate in separate tubes. Relative gene expression was calculated using the 2^−^^ΔΔ^^CT^ method [ 21 ] and normalized to GAPDH expression. The primers used in this study were as follows:

GAPDH (forward, 5′-AGATCCACAACGGATACATT-3′;
  reverse, 5′-TCCCTCAAGATTGTCAGCAA-3′),
α-SMA (forward, 5′-CCGACCGAATGCAGAAGGA-3′;
  reverse, 5′-ACAGAGTATTTGCGCTCCGGA-3′),
CF6 (forward, 5′-TCAGTGCAAGTACCACAGACTC-3′;
  reverse 5′- CACAGAGACTGCTGACCGAA-3′),
and IF-1 (forward, 5′- CGGACTCGTCGGAGAGCAT-3′;
  reverse 5′- TTCTCTCGTTTCCCGAAGGC-3′).

### 2.8. Immunohistochemistry

The right lung lobes were cut and processed as previously described by preparing standard formalin-fixed, paraffin-embedded tissues for HE or regular immunohistochemical staining [ 22 ]. The tissue samples were sectioned at a thickness of 5 μm [ 23 ]. Immunohistochemical analysis involved incubation of the sections with the primary antibody, anti-α-SMA (1:200; Abcam), followed by incubation with a biotinylated secondary antibody, and use of the avidin-biotin complex technique with Vector Red substrate (Vector Laboratories, USA). All images were obtained using an Olympus (Olympus Corporation, Japan) LCX100 Imaging System and analyzed using ImageJ software (version 1.38x; National Institutes of Health) by an expert (L.Y.). In each lung section, small pulmonary arteries (PAs) (50–100 μm in diameter) were analyzed at × 40 magnification in a blinded manner. Medial wall thickness was expressed as the summation of two points of medial thickness/external diameter × 100 (%). Intraacinar (precapillary) PAs (20–30 μm in diameter, 25 vessels each) were assessed for occlusive lesions and rated as follows: grade 0, no evidence of neointimal lesions; grade 1, less than 50% luminal occlusion; and grade 2, more than 50% luminal occlusion [ 24 ]. There was no evidence of neointimal lesion formation in any PAs from control rats (all PAs were grade 0).

### 2.9. Statistics

The data are presented as mean ± standard error of the mean (SEM). The values from the two groups were compared using the unpaired t-test. Analysis of variance (ANOVA) was used to compare multiple groups. Subsequently, the Newman–Keuls test was performed. All analyses were performed using SPSS (version 17.0, SPSS Inc. Chicago, IL, USA). Results were considered statistically significant at p < 0.05.

## 3. Results

### 3.1. Hemodynamic and morphologic changes in hypoxia-exposed PAH rats

**Figure 1:**
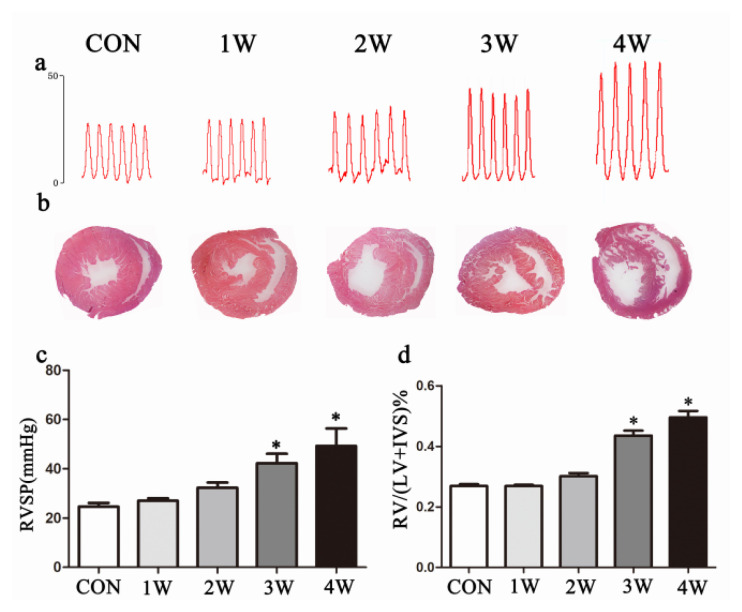
Hemodynamic changes in hypoxia-exposed rats. (A, C) RVSP was significantly increased in the 3- and 4-week exposure groups. (B, D) Rats developed the same elevated right ventricular hypertrophy after 3 weeks of exposure. The data represent the mean ± SEM. *p < 0.05. (RVSP, right ventricle systolic pressure; con, control; 1w, 1-week hypoxia group; 2w, 2-week hypoxia group; 3w: 3-week hypoxia group; 4w: 4-week hypoxia group).

After four weeks of hypoxia exposure, the skin of the rats lost its luster and became very rough, and the degree of dyspnea worsened. Pulmonary vascular remodeling was evaluated by measuring the wall thickness of pulmonary arterioles. After exposure to hypoxia, hemodynamic (Figure 1a) and morphological (Figure 1b) changes were analyzed at 1, 2, 3, and 4 weeks. RVSP was significantly increased in the 3- and 4-week hypoxia exposure groups compared with that in the other study groups. There were no significant changes observed in the 1- or 2-week hypoxia exposure groups relative to the control group (p < 0.05, Figure 1c). The rats also developed the same increase in right ventricular hypertrophy after 3 weeks of exposure in both the 3- and 4 -week exposure group. This was confirmed by the RV/(LV + S) ratio (p < 0.05, Figure 1d).

**Figure 2:**
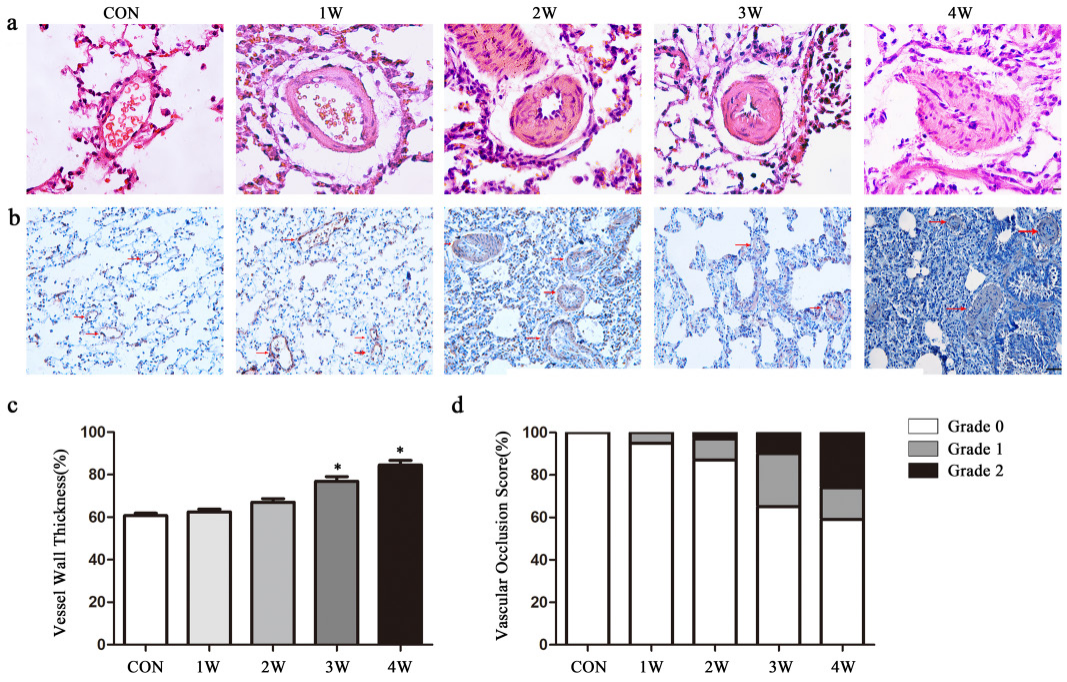
Morphological changes in hypoxia-exposure rats. (A) Hematoxylin and eosin staining. (B) IHC staining for α-SMA antibody. (C) Ratios of vascular medial thickness (i.e. smooth muscle thickness) to the outer diameter (total vessel wall thickness) of small pulmonary arteries (diameter, 50–100 μm) in different groups. (D) Vascular occlusion score of the small pulmonary arteries (diameter, 20–30 μm) in different groups. Data are the mean ± SEM. *p < 0.05 compared with the control group. (con, control; 1w, 1-week hypoxia group; 2w, 2-week hypoxia group; 3w: 3-week hypoxia group; 4w: 4-week hypoxia group).

The same trend was observed in morphological and hemodynamic changes. Hematoxylin-eosin staining indicated that the wall thickness of vessels was significantly increased in the hypoxia group in a time-dependent manner (Figure 2a). Immunohistochemical analysis revealed increased α-SMA immunostaining in the lung tissue of the hypoxia-induced group over time (Figure 2b). The ratios of vascular medial thickness (i.e. smooth muscle thickness) to outer diameter (total vessel wall thickness) of the small pulmonary arteries (diameter 50–100 μm) and the vascular occlusion score of the small pulmonary arteries (diameter 20–30 μm) in different groups also validated PAH (Figure 2c, Figure 2d).

### 3.2. CF6 level in hypoxia-induced PAH

**Figure 3:**
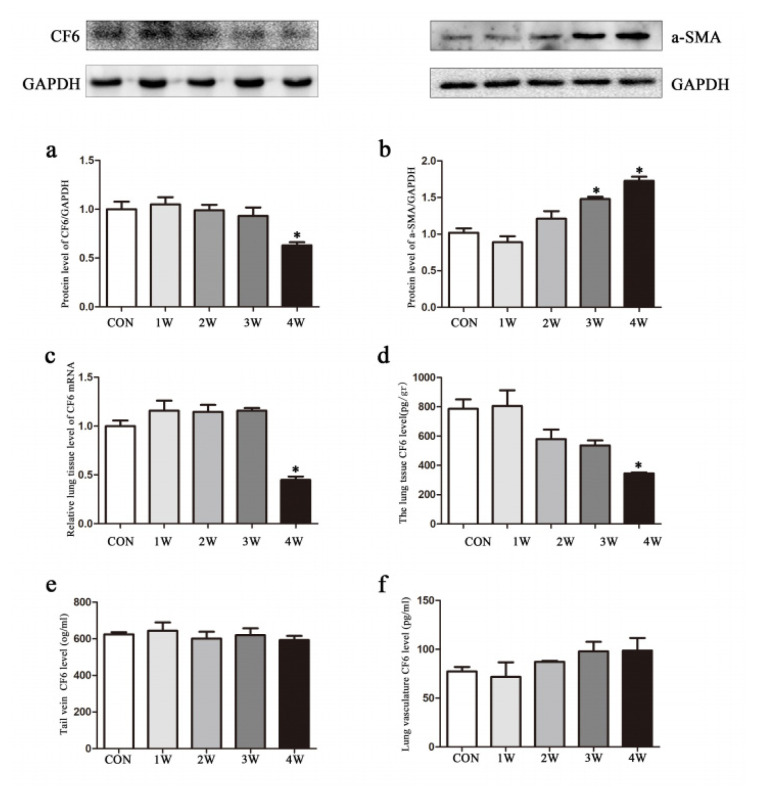
The local expression of CF6 in PAH-induced by hypoxia. The protein level of (A) CF6 and (B) α-SMA were measured in total lung homogenates from PAH rats using western blot. (C) The mRNA level of lung CF6 was measured using RT-PCR. The level of CF6 in lung tissue (D), blood sample in tail vein, (E) and lung vasculature was measured using ELISA. Data are shown as the mean ± SEM, *p < 0.05 compared with control group (con, control; 1w, 1-week hypoxia group; 2w: 2-week hypoxia group; 3w: 3-week hypoxia group; 4w: 4-week hypoxia group).

The expression of CF6 in lung tissue and blood samples from the tail vein of hypoxia-induced PAH rats was examined. Western blotting results for CF6 showed that no significant change was observed in the first 3 weeks, but an almost half-fold change appeared in the 4-week hypoxia group compared with that in the control group (p < 0.05, Figure 3a). Western blot analysis of α-SMA confirmed a 1.7-fold change in CF6 during the development of hypoxia-induced PAH (Figure 3b). Reverse transcription Polymerase Chain Reaction (RT-PCR) was performed to further confirm the mRNA levels of CF6 and showed a significant decrease in the 4-week hypoxia group relative to the control group (p < 0.05, Figure 3c). The level of CF6 in lung tissue was downregulated in a time-dependent manner, and more than a half-fold change was observed in the 4-week hypoxia group relative to the control group (p < 0.05, Figure 3d). The level of CF6 in blood samples from the tail vein (Figure 3e) and the lung vasculature (Figure 3f) was also analyzed to determine its expression. However, there were no significant difference in CF6 levels in blood samples or the lung vasculature in the 4-week hypoxia exposure group compared with those in the control group.

### 3.3. Mitochondrial ATP synthase activity and CF6 level

**Figure 4:**
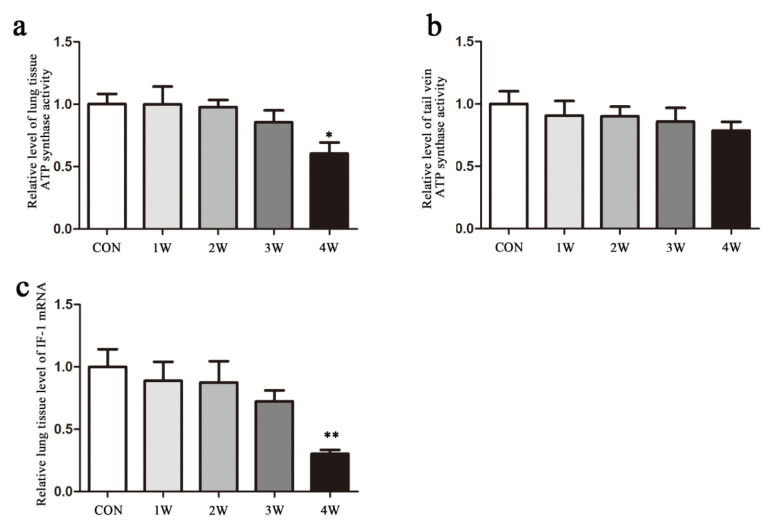
ATP synthase activity expression level in the lung tissue(A) and tail vein blood sample (B) was measured using a microplate assay kit. (C) Expression level of IF1 in lung tissue following hypoxia exposure. Data are shown as the mean ± SEM, *p < 0.05 compared with the control group. Data are shown as the mean ± SEM, *p < 0.05 compared with the control group (con, control; 1w, 1-week hypoxia group; 2w: 2-week hypoxia group; 3w: 3-week hypoxia group; 4w: 4-week hypoxia group).

CF6 is an essential subunit of mitochondrial ATP synthase that is released into the blood to affect vascular function as a circulating vasoconstrictive peptide. Therefore, we analyzed ATP synthase activity to clarify the relationship between CF6 and mitochondrial ATP synthase. The mitochondrial ATP synthase activity in local lung tissue was downregulated in a time-dependent manner and showed a significant decrease only after 4 weeks of hypoxia (Figure 4a). No significant change in ATP synthase activity was observed in the circulatory level in blood samples from the tail vein (Figure 4b).

### 3.4. Local IF1 level in rats with hypoxia-induced PAH

IF1 is a key regulator of ATP synthase and its subunits, such as CF6. To investigate the influence of IF1 in hypoxic conditions, we examined the expression of IF1 mRNA. The IF1 mRNA expression in lung tissue was significantly lower after four weeks of hypoxia and showed a half-fold change in the 4-week hypoxia group relative to the control group (p < 0.05, Figure 4c). This result is consistent with the ATP synthase activity in lung tissue result, but is contrary to the ATP synthase activity in blood samples from the tail vein.[Fig f5-turkjmedsci-52-5-1468]

## 4. Discussion

This study was designed to investigate the role of CF6 in different PAH types. Unlike the increased expression of CF6 in MCT-induced PAH, the expression of CF6 in hypoxia-induced PAH had a different profile. This interesting and unexpected finding demonstrated: 1) in hypoxic PAH, the gene and protein levels of CF6 in lung tissue were significantly lower; 2) no significant change in CF6 levels was observed in blood samples from the tail vein or lung vasculature in hypoxia-induced PAH; and 3) the local mitochondrial ATP synthase activity in the lung tissue showed a significant decrease only after four weeks of hypoxia. No significant changes in mitochondrial ATP synthase activity in blood samples from the tail vein were found.

Significant changes in hemodynamics and morphology confirmed the development of PAH in rats under hypoxic conditions. The expression of α-SMA in the lung tissue of rats exposed to hypoxia was significantly higher, indicating abnormal proliferation of the pulmonary vascular smooth muscle. The etiology of PAH is complex. Hypoxia-induced PAH is characterized by continuously increased pulmonary artery pressure, pulmonary vascular resistance, and RV hypertrophy [ 25 ]. Inflammation and smooth muscle cell proliferation may play key roles in this disease. The hypoxia animal model has been widely used to study PAH [ 26 ]. We successfully established a model of hypoxia-induced PAH, as demonstrated by the increase in RVSP and proliferation of pulmonary arterial smooth muscle cells (PASMCs).

At present, it is unclear whether the increase in CF6 levels in PAH is related to pulmonary constriction induced by PGI_2_ inhibition. Studies have confirmed that CF6 levels are increased in MCT-induced PAH and appear to play an important role in the disease process [ 27 ]. This study found no indication of elevated CF6 levels in hypoxic PAH.

Endothelial cell injury is common in all metabolic diseases and MCT-induced diseases. Circulating levels of CF6 increase after endothelial injury. CF6 is produced in the cytoplasm in an immature form (amino acids 1–108) and released into the extracellular matrix in the mature form (amino acids 33–108) after enzymatic deletion of the signal peptide (amino acids 1–32) [ 28 ]. CF6 can bind to the subunit of ATP synthase, as ectopic ATP synthase, to accelerate proton import and induce intracellular acidosis [ 8, 12 ]. Moreover, CF6 can increase blood pressure and enhance angiotensin II-induced vasoconstriction in small arterioles [ 9, 10 ]. Considerable evidence suggests that CF6 plays an important role in many diseases such as hypertension [ 29 ], diabetes mellitus [ 30 ], acute myocardial infarction [ 31 ], and stroke [ 32 ]. In our previous study, local and circulating levels of CF6 were significantly increased in rats with MCT-induced PAH [ 27 ].

However, this study found no increase in CF6 expression in blood samples from the tail vein or lung vasculature in hypoxic PAH patients. The mechanism underlying hypoxic PAH is completely different from that of MCT-induced PAH. Hypoxic inhalation without direct cell damage showed no increase in CF6 levels. Because CF6 is localized to the surface of endothelial cells and is released mainly by endothelial cells, we can infer that hypoxia-induced PAH initially causes little injury or rupture of endothelial cells. Hypoxia is a strong stress signal, and the first response organ is the intratracheal-lung tissue pathway. The changes in CF6 found in the local lung tissue are rational. Oxygen supply closely influences ATP synthesis, and CF6 is an essential component of the stalk of mitochondrial ATP synthase, and it is also released from the cytoplasm to the cell membrane [ 10, 33 ]. In this study, lung tissue CF6 levels may be modulated by hypoxic stimuli. Furthermore, because of negative regulation by the vasoconstrictor, CF6, we speculate that stress from hypoxia may suppress ATPase and its subunit expression. Our study did indeed indicate a decrease in ATP synthase activity. ATP synthase is a multi-subunit membrane-bound enzyme of the mitochondria that synthesizes ATP via the proton electrochemical gradient produced by the respiratory chain [ 34 ]. The activity of ATP synthase regulates oxidative phosphorylation, and cell death [ 35, 36 ]. Mitochondrial ATP synthase is the fifth and final component of the oxidative phosphorylation chain [ 37 ]. ATP plays a crucial role in adenosine triphosphate (ATP) generation and acts as a chemical fuel for life processes [ 38 ]. However, the mechanism of CF6 decrease in hypoxic lung tissue may require further investigation.

ATPase IF1 is a physiological endogenous inhibitor of ATP synthase. IF1 inhibits both the synthase and hydrolase activity of the ATP synthase [ 39, 40 ]. Walker et al. proposed that IF1 interacts with F1 of the ATP synthase enzyme to inhibit its function [ 41 ]. Many recent studies have indicated that IF1 plays important roles in normoxia, such as the inhibition of cell apoptosis, stabilization of the F1F0-ATPase structure, and inner mitochondrial membrane cristae structure [ 18–20] . In many cancers, IF1 is present at higher levels in the brain, colon, ovary and other organs [ 42–44] . However, the mRNA level of IF1 was downregulated under hypoxic conditions. This result was consistent with the expression of CF6. The codirectional regulation of ATPase-related subunits may allude to the influence of hypoxia on energy metabolism and that CF6 has little effect on hypoxic vasoconstriction.

The limitations of this study are as follows. First, our research lacks three IF1 antagonist interference analyses to confirm the role of IF1 in PAH. Second, other studies on the effect of CF6 in hypoxic PAH in rats must be further investigated. Third, the findings of this study were from rats and could not be projected completely to human beings.

## 5. Conclusions

CF6 showed downregulated expression in lung tissue, but not in the pulmonary vasculature or at the circulation level, in hypoxia-induced PAH. We speculate that hypoxic stress from the intratracheal-lung tissue pathway is different from the MCT-endothelial injury pathway in PAH. This study provides new insights into CF6 expression and the pathogenesis of PAH.

## Figures and Tables

**Figure 5 f5-turkjmedsci-52-5-1468:**
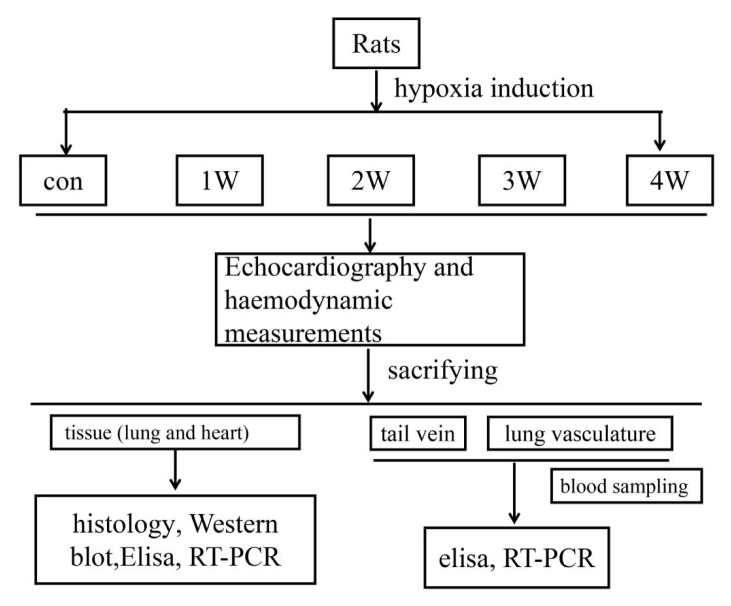
A flow diagram for the experiment steps.

## References

[1] Rabinovitch M Molecular pathogenesis of pulmonary arterial hypertension Journal of Clinical Investigation 2012 122 12 4306 4313 10.1172/JCI60658 23202738 PMC3533531

[2] Benza RL Miller DP Barst RJ Badesch DB Frost AE An evaluation of long-term survival from time of diagnosis in pulmonary arterial hypertension from the REVEAL Registry Chest 2012 142 2 448 456 10.1378/chest.11-1460 22281797

[3] Benza RL Miller DP Gomberg-Maitland M Frantz RP Foreman AJ Predicting survival in pulmonary arterial hypertension: insights from the Registry to Evaluate Early and Long-Term Pulmonary Arterial Hypertension Disease Management (REVEAL) Circulation 2010 122 2 164 172 10.1161/CIRCULATIONAHA.109.898122 20585012

[4] Condon DF Nickel NP Anderson R Mirza S de Jesus Perez VA The 6th World Symposium on Pulmonary Hypertension: what’s old is new F1000 Research 2019 8 10.12688/f1000research.18811.1 31249672 PMC6584967

[5] Nishimura T Vaszar LT Faul JL Zhao G Berry GJ Simvastatin rescues rats from fatal pulmonary hypertension by inducing apoptosis of neointimal smooth muscle cells Circulation 2003 108 13 1640 1645 10.1161/01.CIR.0000087592.47401.37 12963647

[6] Giaid A Nitric oxide and endothelin-1 in pulmonary hypertension Chest 1998 114 3 Suppl 208S 212S 10.1378/chest.114.3_supplement.208s 9741571

[7] Christman BW McPherson CD Newman JH King GA Bernard GR An imbalance between the excretion of thromboxane and prostacyclin metabolites in pulmonary hypertension New England Journal of Medicine 1992 327 2 70 75 10.1056/NEJM199207093270202 1603138

[8] Osanai T Magota K Tanaka M Shimada M Murakami R Intracellular signaling for vasoconstrictor coupling factor 6: novel function of beta-subunit of ATP synthase as receptor Hypertension 2005 46 5 1140 1146 10.1161/01.HYP.0000186483.86750.85 16230521

[9] Osanai T Tanaka M Kamada T Nakano T Takahashi K Mitochondrial coupling factor 6 as a potent endogenous vasoconstrictor Journal of Clinical Investigation 2001 108 7 1023 1030 10.1172/JCI11076 11581303 PMC200946

[10] Osanai T Tomita H Kushibiki M Yamada M Tanaka M Coupling factor 6 enhances Src-mediated responsiveness to angiotensin II in resistance arterioles and cells Cardiovascular Research 2009 81 4 780 787 10.1093/cvr/cvn356 19106112

[11] Asnicar MA Goheen M Bartlett MS Smith JW Lee CH Upregulation of host mitochondrial ATPase 6 gene in Pneumocystis carinii-infected rat lungs Journal of Eukaryotic Microbiology 1996 43 5 38S 10.1111/j.1550-7408.1996.tb04974.x 8822841

[12] Osanai T Kamada T Fujiwara N Katoh T Takahashi K A novel inhibitory effect on prostacyclin synthesis of coupling factor 6 extracted from the heart of spontaneously hypertensive rats Journal of Biological Chemistry 1998 273 48 31778 31783 10.1074/jbc.273.48.31778 9822642

[13] Yin J You S Li N Jiao S Hu H Lung-specific RNA interference of coupling factor 6, a novel peptide, attenuates pulmonary arterial hypertension in rats Respiratory Research 2016 17 1 99 10.1186/s12931-016-0409-5 27491388 PMC4973057

[14] Kilkenny C Browne WJ Cuthill IC Emerson M Altman DG Improving bioscience research reporting: The ARRIVE guidelines for reporting animal research Journal of Pharmacology & Pharmacotherapeutics 2010 1 2 94 99 10.4103/0976-500X.72351 21350617 PMC3043335

[15] Yin J You S Liu H Chen L Zhang C Role of P2X7R in the development and progression of pulmonary hypertension Respiratory Research 2017 18 1 127 10.1186/s12931-017-0603-0 28646872 PMC5483271

[16] Al-Husseini A Wijesinghe DS Farkas L Kraskauskas D Drake JI Increased eicosanoid levels in the Sugen/chronic hypoxia model of severe pulmonary hypertension PloS One 2015 10 3 e0120157 10.1371/journal.pone.0120157 25785937 PMC4364907

[17] Shivshankar P Halade GV Calhoun C Escobar GP Mehr AJ Caveolin-1 deletion exacerbates cardiac interstitial fibrosis by promoting M2 macrophage activation in mice after myocardial infarction Journal of Molecular and Cellular Cardiology 2014 76 84 93 10.1016/j.yjmcc.2014.07.020 25128086 PMC4533121

[18] Bernardi P Rasola A Forte M Lippe G The Mitochondrial Permeability Transition Pore: Channel Formation by F-ATP Synthase, Integration in Signal Transduction, and Role in Pathophysiology Physiological Reviews 2015 95 4 1111 1155 10.1152/physrev.00001.2015 26269524 PMC4600949

[19] Faccenda D Tan CH Seraphim A Duchen MR Campanella M IF1 limits the apoptotic-signalling cascade by preventing mitochondrial remodelling Cell Death and Differentiation 2013 20 5 686 697 10.1038/cdd.2012.163 23348567 PMC3619234

[20] Strauss M Hofhaus G Schroder RR Kuhlbrandt W Dimer ribbons of ATP synthase shape the inner mitochondrial membrane The EMBO Journal 2008 27 7 1154 1160 10.1038/emboj.2008.35 18323778 PMC2323265

[21] Livak KJ Schmittgen TD Analysis of relative gene expression data using real-time quantitative PCR and the 2(−Delta Delta C(T)) Method Methods 2001 25 4 402 408 10.1006/meth.2001.1262 11846609

[22] Alencar AK Pereira SL da Silva FE Mendes LV Cunha Vdo M N-acylhydrazone derivative ameliorates monocrotaline-induced pulmonary hypertension through the modulation of adenosine AA2R activity International Journal of Cardiology 2014 173 2 154 162 10.1016/j.ijcard.2014.02.022 24630383

[23] Kusmic C Barsanti C Matteucci M Vesentini N Pelosi G Up-regulation of heme oxygenase-1 after infarct initiation reduces mortality, infarct size and left ventricular remodeling: experimental evidence and proof of concept Journal of Translational Medicine 2014 12 89 10.1186/1479-5876-12-89 24708733 PMC4022338

[24] Homma N Nagaoka T Karoor V Imamura M Taraseviciene-Stewart L Involvement of RhoA/Rho kinase signaling in protection against monocrotaline-induced pulmonary hypertension in pneumonectomized rats by dehydroepiandrosterone Lung Cellular and Molecular Physiology 2008 295 1 L71 78 10.1152/ajplung.90251.2008 18469113 PMC2494797

[25] Cogolludo A Moreno L Villamor E Mechanisms controlling vascular tone in pulmonary arterial hypertension: implications for vasodilator therapy Pharmacology 2007 79 2 65 75 10.1159/000097754 17148943

[26] Huang X Zou L Yu X Chen M Guo R Salidroside attenuates chronic hypoxia-induced pulmonary hypertension via adenosine A2a receptor related mitochondria-dependent apoptosis pathway Journal of Molecular and Cellular Cardiology 2015 82 153 166 10.1016/j.yjmcc.2015.03.005 25772255

[27] Li N Yin J Cai W Liu J Zhang N Coupling Factor 6 Is Upregulated in Monocrotaline-induced Pulmonary Arterial Hypertension in Rats American Journal of the Medical Sciences 2016 352 6 631 636 10.1016/j.amjms.2016.08.002 27916219

[28] Osanai T Okada S Sirato K Nakano T Saitoh M Mitochondrial coupling factor 6 is present on the surface of human vascular endothelial cells and is released by shear stress Circulation 2001 104 25 3132 3136 10.1161/hc5001.100832 11748113

[29] Izumiyama K Osanai T Sagara S Yamamoto Y Itoh T Estrogen attenuates coupling factor 6-induced salt-sensitive hypertension and cardiac systolic dysfunction in mice Hypertension Research 2012 35 5 539 546 10.1038/hr.2011.232 22258022

[30] Li XL Xing QC Dong B Gao YY Xing SS Plasma level of mitochondrial coupling factor 6 increases in patients with type 2 diabetes mellitus International Journal of Cardiology 2007 117 3 411 412 10.1016/j.ijcard.2006.05.051 16899312

[31] Ding WH Chu SY Jiang HF Cai DY Pang YZ Plasma mitochondrial coupling factor 6 in patients with acute myocardial infarction Hypertension Research 2004 27 10 717 722 15785006 10.1291/hypres.27.717

[32] Osanai T Fujiwara N Sasaki S Metoki N Saitoh G Novel pro-atherogenic molecule coupling factor 6 is elevated in patients with stroke: a possible linkage to homocysteine Annals of Medicine 2010 42 1 79 86 10.3109/07853890903451781 20092401

[33] Osanai T Magota K Okumura K Coupling factor 6 as a novel vasoactive and proatherogenic peptide in vascular endothelial cells Naunyn-Schmiedeberg’s Archives of Pharmacology 2009 380 3 205 214 10.1007/s00210-009-0431-y 19488738

[34] Walker JE The ATP synthase: the understood, the uncertain and the unknown Biochemical Society Transactions 2013 41 1 1 16 10.1042/BST20110773 23356252

[35] Barbato S Sgarbi G Gorini G Baracca A Solaini G The inhibitor protein (IF1) of the F1F0-ATPase modulates human osteosarcoma cell bioenergetics Journal of Biological Chemistry 2015 290 10 6338 6348 10.1074/jbc.M114.631788 25605724 PMC4358270

[36] Boyer PD The ATP synthase--a splendid molecular machine Annual Review of Biochemistry 1997 66 717 749 10.1146/annurev.biochem.66.1.717 9242922

[37] Jonckheere AI Smeitink JA Rodenburg RJ Mitochondrial ATP synthase: architecture, function and pathology Journal of Inherited Metabolic Disease 2012 35 2 211 225 10.1007/s10545-011-9382-9 21874297 PMC3278611

[38] Neupane P Bhuju S Thapa N Bhattarai HK ATP Synthase: Structure, Function and Inhibition Biomolecular Concepts 2019 10 1 1 10 10.1515/bmc-2019-0001 30888962

[39] Garcia-Bermudez J Sanchez-Arago M Soldevilla B Del Arco A Nuevo-Tapioles C PKA Phosphorylates the ATPase Inhibitory Factor 1 and Inactivates Its Capacity to Bind and Inhibit the Mitochondrial H(+)-ATP Synthase Cell Reports 2015 12 12 2143 2155 10.1016/j.celrep.2015.08.052 26387949

[40] Sanchez-Cenizo L Formentini L Aldea M Ortega AD Garcia-Huerta P Up-regulation of the ATPase inhibitory factor 1 (IF1) of the mitochondrial H+-ATP synthase in human tumors mediates the metabolic shift of cancer cells to a Warburg phenotype Journal of Biological Chemistry 2010 285 33 25308 25313 10.1074/jbc.M110.146480 20538613 PMC2919093

[41] Bason JV Montgomery MG Leslie AG Walker JE Pathway of binding of the intrinsically disordered mitochondrial inhibitor protein to F1-ATPase Proceedings of the National Academy of Sciences of the United States of America 2014 111 31 11305 11310 10.1073/pnas.1411560111 25049402 PMC4128166

[42] Wu J Shan Q Li P Wu Y Xie J ATPase inhibitory factor 1 is a potential prognostic marker for the migration and invasion of glioma Oncology Letters 2015 10 4 2075 2080 10.3892/ol.2015.3548 26622799 PMC4579847

[43] Zhang C Min L Liu J Tian W Han Y Integrated analysis identified an intestinal-like and a diffuse-like gene sets that predict gastric cancer outcome Tumour Biology 2016 10.1007/s13277-016-5454-7 27858295

[44] Sanchez-Arago M Formentini L Martinez-Reyes I Garcia-Bermudez J Santacatterina F Expression, regulation and clinical relevance of the ATPase inhibitory factor 1 in human cancers Oncogenesis 2013 2 e46 10.1038/oncsis.2013.9 23608753 PMC3641363

